# Complete Genome Sequence of the Porcine Epidemic Diarrhea Virus Variant Tottori2/JPN/2014

**DOI:** 10.1128/genomeA.00877-15

**Published:** 2015-08-13

**Authors:** Satoshi Murakami, Ayako Miyazaki, Osamu Takahashi, Wataru Hashizume, Yoichi Hase, Seiichi Ohashi, Tohru Suzuki

**Affiliations:** a Thermo Fisher Scientific, Life Technologies Japan Ltd., Tokyo, Japan; b Viral Diseases and Epidemiology Research Division, National Institute of Animal Health, NARO, Ibaraki, Japan

## Abstract

Porcine epidemic diarrhea virus (PEDV) is a cause of diarrhea outbreaks at swine farms, causing vomiting, severe diarrhea, and mortality in piglets. We sequenced and analyzed the complete genome of recently isolated strains. Tottori2/JPN/2014, one of the sequenced PEDV strains, had a unique large deletion in the S gene.

## GENOME ANNOUNCEMENT

Porcine epidemic diarrhea (PED) was first reported in the United Kingdom of Great Britain and Northern Ireland in 1971 (1). Since then, outbreaks have occurred in Europe, Asia, the United States, Canada, and Mexico, with recent outbreaks occurring in 2013 (2–6). PED is caused by PED virus (PEDV), a member of the genus *Alphacoronavirus* (family *Coronaviridae*, order *Nidovirales*). PEDV is a single-stranded positive-sense RNA virus that causes an acute and highly contagious enteric disease characterized by severe enteritis, vomiting, and watery diarrhea in swine (6–8). In October 2013, outbreaks of PED reemerged in Japan for the first time in 7 years. From October 2013 to December 2014, PED occurred at 837 farms throughout Japan, causing death in about 390,000 head of swine, mainly piglet, as reported by the Ministry of Agriculture, Forestry and Fisheries (http://www.maff.go.jp).

In October 2014, a unique case of PED, associated with no mortality rates among piglets, was observed in Tottori Prefecture. We developed a system to analyze the entire genome of PEDV (unpublished data). Briefly, RNA from the PEDV strain Tottori2/JPN/2014 was reverse transcribed and PCR amplified using specifically designed primer sets against the PEDV genome. The amplified genome was sequenced using next-generation sequencing technology on an Ion PGM platform (Life Technologies, Carlsbad, CA, USA) according to the manufacturer’s instructions. The data were assembled using Torrent Suite 4.2 based on known PEDV sequences, such as USA/Colorado/2013 (GenBank accession no. KF272920) (9). The complete genomic sequence of the Tottori2/JPN/2014 strain was found to be 27,342 nucleotides (nt) in length, shorter than that of USA/Colorado/2013 (28,038 nt), with no changes other than a large deletion (582 nt) in the S gene and the presence of unidentified regions in both the 5′ and 3′ terminals. Comparative sequence analysis demonstrated that the genome of the Tottori2 PEDV strain had 99.73% to 99.87%, 99.64% to 99.69%, and 98.90% to 99.45% nt identities with those of North American clades I and II and U.S. S indel PEDV strains used in the phylogenetic analysis, respectively (6). Phylogenetic analysis of the S genes demonstrated that the Tottori2 PEDV strain had the closest genetic relationship with the U.S. PEDV strain TC-PC177 (97.89% nt identity), exhibiting a large deletion in the S gene derived from cell adaptation (10). A phylogenetic dendrogram was constructed using the entire sequence data without consideration of gaps. The results indicated that the Tottori2 PEDV strain was classified into a cluster of North American clade I and was most closely related to the U.S. PEDV strain Iowa103 (11).

These data are expected to facilitate analyses of the epidemiology and evolutionary characteristics of PEDVs in Japan.

### Nucleotide sequence accession number.

This whole-genome sequence of PEDV variant Tottori2/JPN/2014 has been deposited in GenBank under the accession no. LC022792.
